# Analyzing Municipal Solid Waste Treatment Scenarios in Rapidly Urbanizing Cities in Developing Countries: The Case of Dar es Salaam, Tanzania

**DOI:** 10.3390/ijerph16112035

**Published:** 2019-06-07

**Authors:** Emmanuel Kazuva, Jiquan Zhang

**Affiliations:** 1 School of Environment, Northeast Normal University, Jilin Province, Changchun 130024, China; yim124@nenu.edu.cn; 2 Department of Geography, FASS, Open University of Tanzania, Dar es Salaam 23409, Tanzania; 3 Key Laboratory for Vegetation Ecology, Ministry of Education, Changchun 130024, China

**Keywords:** municipal solid waste, environmental cost, economic cost, scenarios, multi-criteria analysis, treatment options, ELECTRE, Dar es Salaam

## Abstract

Currently, large quantities of municipal solid waste (MSW) in many cities of the developing countries are being dumped in informal or formal but unregulated dumpsites that threaten the ecological environment and general public health. The situation in Dar es Salaam, Tanzania is of particular concern and is further challenged by a rapidly growing population and urbanization without adequate waste management systems. Current MSW treatment options have been selected based on the judgment and the experience of individuals with authority while underestimating the role of scientifically derived techniques. This study analyzes the most efficient waste treatment options, particularly scenarios with the lowest economic and environmental costs (EcC and EnC, respectively). It uses 12 years (2006–2017) of MSW management data and compares potential waste treatment options for the identified waste streams. A total of 108 different scenarios were designed, and a multi-criteria analysis method was applied to enable the identification of 11 scenarios with acceptable EcCs and EnCs. These formed an initial decision matrix of aggregation dominance that was then categorized into four groups, each represented by the most ideal point. Finally, the dominant scenario that formed the core for all considered options was found. It costs around $274,100 USD while saving about 1585 metric tons (MT) of CO_2_ emissions daily. This suggests that after all the MSW generated in the city is collected and segregated, organic waste should be composted whilst plastic, paper, glass, and ferrous metal should be recycled. After treatment, other waste will go to some form of landfill. Sustainable management of MSW in this city and others with similar conditions should consider particular local conditions and could use the methods and the findings of this study as a starting point.

## 1. Introduction

Increases in human population, urbanization, economic growth, and associated consumption patterns have significantly added to the volume of materials discarded as municipal solid waste (MSW) [1,2]. From an optimistic perspective, increased volumes of solid waste are not considered a problem; rather, they are considered to provide free resources and employment opportunities, especially for the poor and the marginalized [3,4,5]. Under this perspective, a focus on improving infrastructure, applying modern technologies, and using the best management approaches from a list of scientifically derived options [6] ensures sustainable management of MSW [7].

However, from a pessimistic viewpoint, MSW is a problem causing pollution and other related environmental disasters. By this perspective, additional waste volumes jeopardize the sustainability of the ecological environment and human health [8,9]. This perspective is most evident in developing countries, as their waste management resources are more limited than those in developed countries [10]. In developing countries, MSW is considered a major urban pollutant, posing risks to the environment and public health [10,11]. For example, a study on MSW management challenges in developing countries, which used Kenya as the case study, indicated that over a period of 22 years, collected waste was always less than 40% (on average) of the total waste generated in Nairobi. MSW is disposed of in open dumps that lack proper environmental pollution control and monitoring. In that study, poor MSW disposal was found to be the main source of pollution in many urban centers [12]. In Nepal, only 35–55% of the total waste generated is collected [13]. Similar studies conducted in diverse developing countries (including Ethiopia [14], Vietnam [15], Iran [16], and India [17]) generally indicate that a lack of technology, poor infrastructure, and financial capacity are the leading challenges for inadequate MSW management (MSWM).

While the trend in MSW generation is increasing continuously worldwide, and the related pollution is becoming a serious environmental concern, several governments are making efforts to reduce the problem [12,18,19]. However, the effectiveness of these efforts, particularly in Africa, are limited by a number of challenges, including a lack of necessary infrastructure and monitoring capacity [15,17,20,21]. Tanzania, as with other developing countries, is concerned about dealing with the high level of urban pollution from MSW [11,18]. Dar es Salaam, the largest and the main commercial city in the country, is confronted with all these challenges as well as high population and rapid urbanization, all of which lead to inadequate responses from responsible authorities [11,22]. According to the last official national population and housing census, the population of Dar es Salaam was 4.4 million in 2012, [23] but was an estimated 6.4 million according to the world population projections for 2019 [24]. Currently, the environmental risk index (ERI) for this city is at the medium level (0.4–0.6); however, it has been on a continuous upward path for the last 10–15 years. Indexes such as “pressure” and “state” are at relatively high levels (>0.6), while the index “impact” is medium (0.59) but also approaching a high level. Therefore, the actual state of the environment is not appealing, and with the influence of the rate of population and urbanization increase, the situation may be worse in the near future [11].

Managing MSW requires appropriate financial resources, infrastructure, and technology. However, in Tanzania, as in other developing countries, the application of technology for MSWM is very limited [18,22]. In this case, strategic MSWM planning at all levels is essential [16,25]. Strategic planning aids viable decision-making in waste management schemes. It takes into account the adverse environmental, economic, and general social impacts that may result from inadequate MSWM and treatment options [26]. A strategic plan assists in the proper allocation of limited resources [27]. Decisions on which method and/or approach to use are tailored to the “specific social-demand” by comparing and selecting the best options from a list of identified alternatives [28,29].

While MSWM is complex, the level of complexity varies according to the waste streams and their characteristics. Because of this complexity, expertise is required for the identification of waste types, tools for segregation, transportation routes, and treatment and final disposal methods. All these need to be addressed in a coordinated manner [30], thus necessitating a sensible decision-making process that deliberately analyzes waste management approaches that respond to physical, chemical, and biological conditions in a particular area and for particular waste streams [31,32].

Many governments of developing countries adopt the waste management scenarios of developed countries derivatively with little or no regard for their suitability in the particular local environment [32,33,34]. Most adoptive projects fail to properly consider their economic implications and environmental consequences [12,20,35,36]. Thus, such projects either fail at the initial stages of implementation or, if they manage to take off, affect the environment and public health negatively. To avoid this, it is important to establish a reliable method for MSWM using established numerical standards that assist in defining a specific set of possible alternatives. The choice of which approaches to take must be guided by an astute model considering social demand and including, in particular, the environmental and the economic costs [28,37].

Therefore, this study reviews potentially desirable scientific approaches to dealing with the waste problem in the rapidly urbanizing cities in developing countries. It analyzes different waste management scenarios with respect to the actual situation in Dar es Salaam by using the multi-criteria analysis method—the Elimination and Choice Expressing Reality (ELECTRE) method—which uses a process of elimination from a list of developed options until the best choice is reached based on specific local demand and capacities [38,39]. The study provides results that may also be applicable in other cities of similar characteristics and proposes the best scenarios that met the criteria of minimum economic and environmental costs while considering technological ease and level of social acceptance.

## 2. Materials and Methods

For ease of analysis, both basic and technical data were sought for the study. The methodology was divided into two major stages; namely, (1) basic data acquisition and (2) scenario development.

### 2.1. Basic Data Acquisition

At this stage, basic data relevant to MSWM in Dar es Salaam was sought. Aspects considered included waste generation level and management status of different waste streams in the period 2006–2017, which was deemed an acceptable “environmental pollution risk assessment period” [40,41,42,43]. Other data were related to risk prevention, protection, and mitigation measures such as waste management systems, population density, settlement patterns, urbanization level and trends, and human factors such as waste management experience and worker skillsets. For population projection, the study used national population data from the National Bureau of Statistics (NBS) [44], as reported in the previous work by Kazuva et al. [11].

To determine per capita generation rates and subsequent projections, we used data obtained from the four sampled wards, Mikocheni, Oyster-bay, Mwananyamala, and Buguruni, which had a total of about 187,000 inhabitants. The selected wards vary in their economic character and their settlement patterns. The former two wards represent the suburbs—areas with properly planned and serviced locations. They include the homes of some major political figures, government officials, and some merchants. The latter two wards are among low-income neighborhoods characterized by poor settlement planning, low-quality housing, and limited social services [45]. Working in these areas, we obtained the average total waste generated per day and then used population data to determine the overall per capita MSW generation rate to be 0.80 kg/capita/day. This was consistent with a previous study by a 2016 Expert Mission on integrated solid waste management (ISWM) from Dar es Salaam, which estimated waste generation to be 0.82 kg/capita/day [18].

Understanding the composition of MSW is an important aspect of developing different treatment and management scenarios. However, this was not an easy task in the city of Dar es Salaam, where waste segregation is very low and recycling systems are poor. The available literature indicates that the composition of MSW varies greatly from one municipality to another and changes significantly over time [22,46,47,48]. However, we managed to estimate the composition of MSW generated in Dar es Salaam from the surveyed area. In this case, each ton of MSW was composed of 57.21% organic waste, 13.08% plastic waste, 6.12% paper-related waste, 2.32% glass, 1.02% ferrous metal (steel and aluminum), and 20.25% other waste components [18]. This information was particularly important for scenario development.

### 2.2. Scenario Development

This stage was fundamentally important for identification of the ideal conditions for MSWM in Dar es Salaam. In this stage, all possible waste treatment methods were analyzed against each waste type (waste stream), and selections were made based on the qualities of a particular option. The stage involved two steps—scenario design and scenario selection.

#### 2.2.1. Scenario Design

In designing waste treatment scenarios, waste composition was an important consideration, as each stream has its own suitable treatment methods [49,50]. For instance, the methods that are preferable for organic home-based waste are seldom used for metal or electronic waste, and vice versa [51,52]. Six major waste streams—namely, organic, plastic, paper, glass, metal, and others (one group representing all other remaining MSW)—were identified. Each stream was thoroughly examined considering five potential treatment options: composting, recycling, incineration, pyro-gasification, and sanitary and/or bioreactor landfilling. These were numbered 1, 2, 3, 4, and 5, respectively. For the effective organization of all scenarios and ease of analysis and selection, the waste categories/streams were also placed in a precise order, which was used throughout the process [53]. Therefore, the six identified waste streams in the order mentioned above were considered relative to the named treatment options (Table 1). The symbol ‘‘+’’ stands for a viable treatment option, and ‘‘−’’represents a non-viable option.

Table 1 shows each stream with its potential MSW treatment options based on expert inputs and comprehensively reviewed literature from reputable sources. All scenarios (see Section 3.2) were identified and given different codes for computational purposes [49,54]. For instance, the codes for the first and the last scenarios (S1 and S108) were 122223 and 555525, respectively. From S1, this means that organic waste must be composted through windrow, vermicomposting, or another suitable composting form, as signified by the first digit of its code (1); plastic and rubber waste, paper waste, glass, and ferrous metal must be recycled, as signified by the second, the third, the fourth, and the fifth digits in the code (2); and other wastes must be incinerated, as shown by the sixth digit of the code (3). The last scenario proposes that all waste streams except metal go to sanitary or bioreactor landfill (5). Metal is suggested for recycling, as signified by the fifth digit of this code (2). All scenarios used this coding system.

#### 2.2.2. Scenario Selection

Selecting the best option for handling MSW from a long list of available alternatives is a complex activity that demands a critical investigation based on the particular advantages and the limitations of each scenario [31]. The complexity is more significant when some of the options are drawn from different localities [55,56]. To make the selected scenarios useful for decision-making and to provide timely demand-specific solutions, it is important that the selection process considers social-cultural, economic, environmental, political, and legal aspects of the specific area in question [57,58]. This study considered these diverse factors, giving special attention to economic cost (EcC) and environmental cost (EnC) for all the scenarios formulated. However, the unavailability of reliable financial and environmental data from the study site was one of the main limitations of this study. Many countries do not make financial and environmental cost figures accessible in the public domain [59]. Both public and private sectors involved in MSWM may lack such important data or, if they have them, may be reluctant to disclose any that could be considered confidential [56]. However, as the provision of a clean environment (including waste management services) is a human right and a proxy of good governance and the sound management of public funds, making such data available for research and other uses should be standard practice [60,61,62].

Despite the lack of complete financial and environmental data, we used basic information available from reliable sources, such as the Vice President’s Office (VPO), the National Environmental Management Council (NEMC), and Dar es Salaam Local Authorities (DLAs), to compute economic and environmental costs. The information was obtained through different methods, including an inventory survey whereby information including the cumulative amount of MSW generated and the percentage composition of each waste stream in Dar es Salaam from 2006–2017 was estimated.

**Figure 1:**
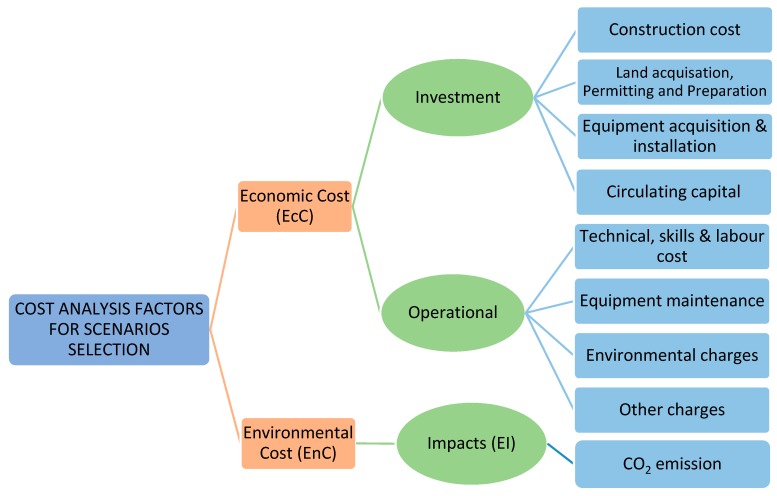
Ideal factors for scenarios selection.

For effective analysis of EcC, aspects such as the initial investment in a particular technology and the unit operating costs of treatment facilities need to be assessed [59,63] as shown in Figure 1. However, within EcC, this study considered only operational costs, e.g., technical skills and labor cost, equipment maintenance, environmental charges, and other charges.

Because reports of EcC for different waste treatment options were insufficient, in some cases, researchers used secondary data from in-depth reviews from both developed and developing countries with similar experiences. This helped to generate an average unit cost (expressed in USD) for treating 1 metric ton (MT) of MSW for all treatment options in each waste stream, as shown in Table 2. This made it possible to estimate the entire EcC for the total amount of MSW generated in Dar es Salaam.

The EnC was calculated mainly based on the carbon emissions from MSW treatment. This considered the total waste generated in Dar es Salaam and applied a waste carbon calculator (WCC) to determine net carbon emissions in MT of CO_2_ equivalent, as adopted from the University of Texas (Table 2). This was supplemented by emissions factors provided by the CA-CP Campus Carbon Calculator, now administered and maintained by the Sustainability Institute at the University of New Hampshire. The estimated EnC for treating 1 MT of each waste stream was obtained from this analysis (Table 2).

To obtain the total economic and environmental costs, the average cost calculated for treating 1 MT of each waste stream (Table 2, Sections “A” and “B”) was multiplied by a total number of tons of a particular stream produced per day.

Given that waste generation for the entire assessment period (2006–2017) showed a continuous upward trend with no sign of decline, which was also supported by the linear projection (Section 3.1), it was considered appropriate to use data from the last year of assessment (2017) to compute both EcC and EnC. The method is robust; should there be any changes in volumes or trends of generated waste, such changes can easily be accommodated by making adjustments based on the new figures.

Apart from other factors such as the recovery of electricity under some scenarios [31], the criteria for scenario selection was mainly based on the two factors.

#### 2.2.3. Using the Elimination and Choice Expressing Reality (ELECTRE) Method for Scenario Selection

The ELECTRE method is one of the multi-criteria analysis approaches first proposed by Bernard Roy and his colleagues at the consultancy firm SEMA and used to choose a new activity from the list of available options. It ranks relations using value comparisons separately among alternatives under each one of the criteria [87,88,89,90]. This method particularly suits decisions about complex environmental problems such as climate change [91] and urban pollution, including MSWM [38,92].

Analyzing economic and environmental costs for scenario selection was the core objective of this study. A weight vector (*w*) was subjectively assigned to simplify this analysis and ease the data processing and handling. An ELECTRE algorithmic structure was used to set a matrix and define *w* for each economic and environmental value. In this case, each factor was given a half weight (50%). Scholars are concerned about the sensitivity of choices to the assigned weights, which normally depend on an expert’s point of view [31,93]. In this study, the effect of variations in weight (EcC and EnC) was minimal and was found to have no impact when analyzing indices. Therefore, variations in value were negligible, and the final matrix of aggregate dominance was identified robustly.

Several operational steps in the application of the ELECTRE method have been highlighted by different scholars. These can vary according to the specific purpose. Despite these differences, there are three major common steps; namely, normalizing the decision matrix, weighting the normalized decision matrix, and determining the concordance and the discordance [87,94,95]. However, for ease of analysis, the three steps can be subdivided into several smaller steps [93,96,97]. This study employed eight steps to apply ELECTRE: (1) organizing elements for initial computation, (2) standardizing elements of the initial decision matrix, (3) weighing the standardized decision matrix to construct outranking relationships, (4) constructing matrices of concordance and discordance, (5) constructing a discordance index, (6) setting concordance and discordance thresholds, (7) constructing matrices of concordant and discordant dominance, and (8) constructing a matrix of aggregate dominance. To reach the desired goal when using the ELECTRE method, each step was considered important [93] and was followed systematically using the general ELECTRE equation given below:

(1) $$X_{g}=\frac{a_{g}}{\sqrt{\sum_{i=1}^{M}a_{g^{2}}}}$$

Since each developed scenario represents two analyzed values, they were presented in a Cartesian coordinate system using the *Y* and the *X* axis for economic and environmental values, respectively. From the graphical representation, acceptable scenarios were easily observed based on the criteria set, i.e., EcC ≤ 3 × 10^5^ USD and EnC ≤ 360 metric tons of CO_2_ emission (see Section 3.2).

The development of the matrix of aggregate dominance provided researchers with the best options from a list of 108 scenarios. This was an important first step in decision-making. Many attempts to reduce environmental pollution from MSW in cities in developing countries fail because of high economic costs [98]. This had also been noted in Dar es Salaam [22,48,99]. It was, therefore, important to make sure that the dominance matrix from which the best scenarios were drawn was appropriate not only in terms of the environmental quality sought, as obligated by the international community, but also in terms of the economic capacity of the country. In this way, the study findings could produce economic benefits and support a sound environment quality for current and future generations in Dar es Salaam and other cities with similar characteristics [59,84,86].

Therefore, scenarios that met the conditions set, i.e., a daily cost of EcC ≤ 3 × 10^5^ USD and EnC ≤ 360 metric tons of emitted CO_2_, were selected. The scenarios were thus divided into two sets—acceptable if they met both criteria and unacceptable if they failed to meet one or the other criterion. The sets of acceptable scenarios formed the initial decision matrix. Using the classification guide shown in Table 3, the acceptable scenarios were categorized to support further shortlisting of preferred options (also see Section 3.3 and Section 3.4).

The acceptable scenarios were then categorized into four major groups based on their similarities and differences, and a scenario with the minimum EnC was selected from each group to be described and used in final decision making.

## 3. Results and Discussion

### 3.1. Generation Trend of MSW in Dar es Salaam

As Table 4 shows, the rate at which MSW was being generated in Dar es Salaam increased significantly for the last 12 years; responses were inadequate. This trend made the environmental risk index (ERI) rise from 0.3–0.5 (risk level) from 2006–2017. More importantly, some indices such as pressure, state, and impact were more worrying than the comprehensive ERI [11].

**Figure 2:**
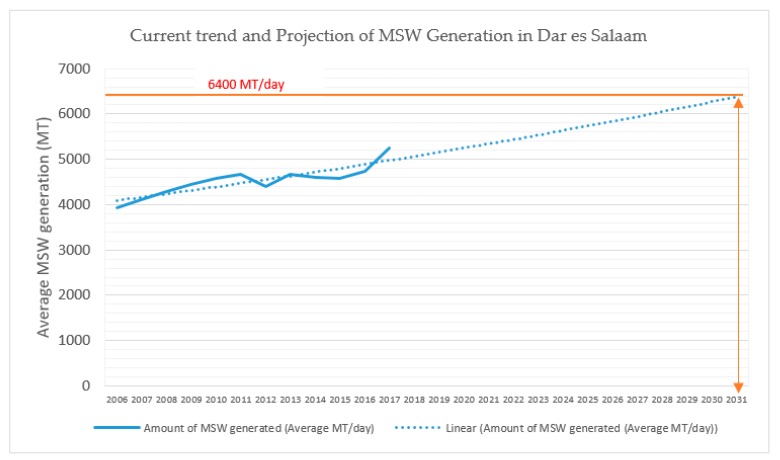
Trend and projection in municipal solid waste generation.

Based on the actual and the projected situation, it is evident that MSWM in this area is problematic and threatens the ecological environment and human health. As clearly shown in the national population projection report for 2013–2035 [44], the rate of MSW generation has increased over the years. If nothing is done to improve the current management situation, this trend will worsen in the future, and the environmental quality will deteriorate further [11]. To confirm this, Figure 2 shows a fitted linear trend line (R^2^ = 0.44) for MSW generation based on the following equation:

*Y* = 80.339*x* + 3999.9 (2)

where *x* is the year.

This means that MSW generation in Dar es Salaam increased by approximately 80 tons every year from 2006–2017. If this trend does not change (and it is likely to increase), it is estimated that by 2031, the amount of MSW generated will be more than 6400 MT per day (Figure 2).

The results above were consistent with the previous study by Kazuva et al. [11] which showed that, under the impact of urbanization, improving standards of living (tending to change consumption patterns), and other driving forces, MSW generation will rapidly increase and raise the environmental hazard level, with ERI rising to or near the critical point (≥0.8). If there is no prompt and effective response from the current management, MSW will become a major threat to the ecological environment and public health in this city.

### 3.2. Formulated Scenarios for MSW Management in Dar es Salaam Code

As noted, all possible combinations of potential MSW management and treatment options for all waste streams in Dar es Salaam were quantified. As a result, a total of 108 scenarios were obtained, as presented in Table 5. Each scenario represents a complete waste treatment option, considering the differences in the percentage composition of each stream (see Table 4 and the description of the scenario development stage (Section 2.2.1)).

From the set conditions as stated in the Methodology section, all these scenarios were analyzed using economic and environmental variables. The intention was to make sure that any selected scenario is not only economically efficient by considering the local economy but also does not compromise environmental quality for current and future generation. EcC and EnC were computed for all scenarios and, as shown in Table 6, the maximum costs were $512,500 USD (EcC) and 3825 MT of emitted CO_2_ (EnC) per day, which occurred in scenarios S70 and S89, respectively. In contrast, the minimum cost was $211,800 USD (EcC) and −3546 MT of CO_2_ emissions (EnC) per day for S2 and S55, respectively.

**Figure 3:**
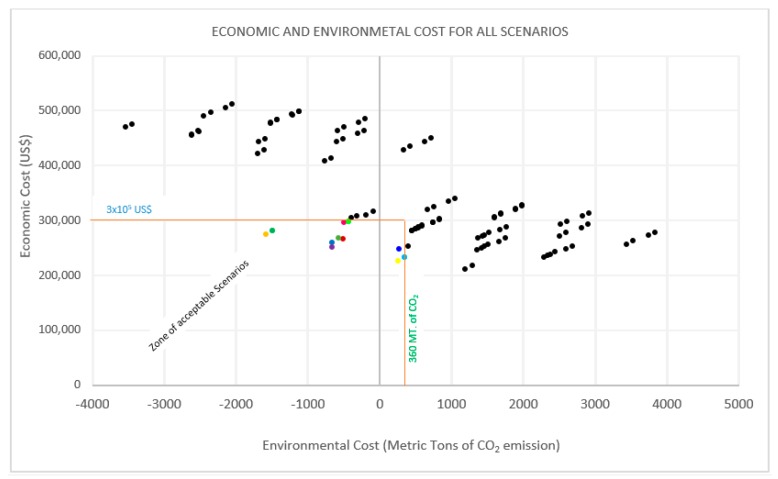
Graphical representation of economic and environmental costs for all scenarios.

Figure 3 is a graphical presentation of the unit costs (EcC and EnC) of all scenarios. Scenarios with the lowest economic and environmental cost fell within the “zone of acceptable scenarios”. They were clearly identified and confirmed to have met the set criteria for initial selection.

### 3.3. Selected Scenarios for Initial Decision Matrix

As indicated in Figure 3, scenarios that met the set criteria, i.e., EcC ≤ 3 × 10^5^ USD as the daily cost of the comprehensive treatment of all MSW in the city and an EnC ≤ 360 metric tons of emitted CO_2_, were selected for the next stage of analysis.

However, the action plan toward implementation of goal 13 (climate action) of the 2030 sustainable development goals (SDGs) and the International Organization for Standardization (ISO) standards imply that any development project should include initiatives that reduce greenhouse gas (GHG) emissions to the atmosphere [85,100,101]. The MSWM scenarios should be considered in light of this obligation. In this regard, the most favorable option (the ideal point) would be the one with the lowest environmental cost. From the scenarios generated (Figure 3), this would be S55 (−3546.30 MT CO_2_ emissions). However, this study also considered the economic costs. S55 had a very high EcC (approximately $470,200 USD per day), which is not favorable given the current economic status of this country and other developing countries [31,102,103,104]. Therefore, this scenario would not be acceptable in the Dar es Salaam environment. As Figure 3 shows, there were a number of other scenarios with good environmental consequences (S58, S57, S60, and S61) that were outside the acceptable economic cost boundary.

On the other hand, if selection were to be based merely on economic efficacy, the ideal scenario would be S2, which costs only $211,800 USD per day. However, from the United Nation (UN) and ISO action plans, this scenario was not acceptable, as its EnC (1189.74 MT CO_2_ emission) was high compared to other scenarios. Other scenarios with this behavior (economic efficiency but high environmental cost) included S5, S8, and S11. Choosing one of these to deal with MSW would pose a serious environmental risk by increasing GHG emissions (in this case, CO_2_), jeopardizing the ecological environment and human health [105]. Indeed, most of them were outside the acceptable scenarios selection zone (Figure 3). Therefore, after all the analysis, eleven scenarios (S19, S22, S21, S1, S24, S4, S25, S91, S20, S3, and S23) (highlighted in Figure 3) were found to have met the minimum requirements and formed the initial decision matrix. These scenarios and their EcCs and EnCs are listed in Table 7.

### 3.4. Grouping of Acceptable Scenarios

From the initial acceptable points, any scenario could be selected. However, further analysis was needed to reach the most favorable options with both competitive advantages (EcC and EnC). Furthermore, the selected scenarios had some common characteristics, allowing them to be classified into groups based on their similarities and their differences by the use of the acceptable scenarios classification guide (Table 3). Four major groups were identified using the ELECTRE method: (1) the most favorable scenarios, (2) favorable scenarios, (3) less favorable scenarios, and (4) intermediate scenarios (Table 8). As explained in the Methodology section, each group could be represented by one scenario; that is, the most favored scenario in the group was that with the minimum environmental cost. In each group, this representative scenario was used for further analysis, description, and final decision making.

**Figure 4:**
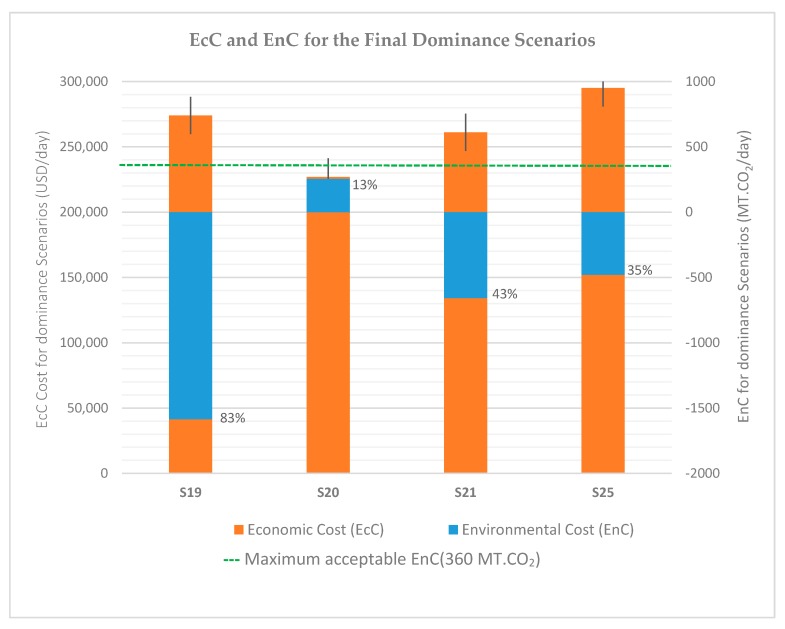
The Elimination and Choice Expressing Reality (ELECTRE) aggregates of dominance scenarios.

Any scenario at this level was considered economically and environmentally acceptable. In this case, the environmental cost was given the first priority to determine the acceptability level of each point. The ELECTRE aggregate of dominance matrix is graphically presented in Figure 4, indicating four scenarios as the final selected ideal points (S19, S21, S25, and S20). Each point was carrying two analyzed economic and environmental factors. As shown in the figure, the longer the EnC value went far below the “maximum acceptable environmental cost level”, the more the scenario became suitable for adoption. Thus, the level of acceptance for each case was identified, whereby S19 had the highest dominance degree (83%) followed by S21 (43%), S25 (35%), and S20 (13%).

The level of acceptance for all scenarios must reflect the actual situation with MSW in Dar es Salaam. This includes socio-economic and environmental aspects, population trends, future waste generation projections, available national policies, and international agreements on the environment. Therefore, with the objective of sustainably managing and treating MSW in Dar es Salaam, the four favored scenarios are considered in more detail below.

The first option (S19) was the most favored option with the highest acceptance level (Figure 4), costing $274,065.03 USD and −1585.02 MT of CO_2_ emission per day. According to the classification guide, the scenario had a low EnC and a medium EcC. It was a ‘‘most favorable option’’ (Table 8). The scenario suggests that after all the generated MSW in Dar es Salaam is collected and segregated, organic waste would be composted, whilst plastic, paper, glass, and ferrous metal would be recycled. The remaining waste, consisting of 20.25% of the total, would go to sanitary and/or bioreactor landfills. To a great extent, this scenario was consistent with the previous work by the ISWM to Dar es Salaam [18] and other scholars that have worked on MSWM in this city [48,99,106].

The second option (S21) had costs of $261,052.80 USD and −657.24 MT of CO_2_ emission per day. The scenario was similar to S19 but differed in that plastic waste would go to sanitary and/or bioreactor landfill rather than recycling. This would somewhat increase CO_2_ emissions (an addition of 1.35 MT of CO_2_ per ton of MSW treated) but represented a reduction in EcC of approximately $30 USD for every ton of plastic waste compared to the first option (S19). Therefore, despite being more economically competitive than S19, the additional GHG emissions made it not favored for the first position.

The third option (S25) required that organic waste be composted, while paper waste would be incinerated for energy recovery. The other waste streams (plastic, glass, and ferrous metal) would be recycled, while the remaining waste ‘’others’’, as in the previous options, would go to sanitary and/or bioreactor landfills. The costs were $295,045.74 USD/day and −489.97 MT of emitted CO_2_. It was clear that this scenario was significantly less favorable in both economic and environmental cost compared to the previous two options. It had an advantage in the speed of implementation, as it involved the use of more sophisticated technology. Therefore, its adoption could reduce the human health-related impacts from direct contamination. However, it remained the third choice.

The fourth option (S20) had characteristics distinct from the other favored scenarios. Among the four, it had the lowest EcC ($225,908.77 USD/day) but the highest EnC (260.21 MT of CO_2_). The scenario required that organic waste be composted while plastic waste be incinerated. The other waste streams (paper, glass, and ferrous metal) would be recycled, while ‘’others’’ waste would go to landfill. Despite this scenario having the lowest EcC (mainly because the incineration of plastic waste increases the rate of return from energy recovery), this treatment option would not only add to the amount of GHG emitted but would also lead to ashes and dust being deposited in surrounding areas, posing a risk to the ecological environment and general public health [107,108,109]. Because of this, the scenario was the least favored of the acceptable scenarios.

Although each of these four scenarios had independent features, they all included composting of organic waste as the best treatment option due to its economic advantages. However, landfills have been the main option used for the treatment of organic waste in the city of Dar es Salaam. Scholars indicate that the sites used as landfills are usually changed into uncontrolled dumpsites [18,110]. Therefore, this study recommends the adoption of organic waste composting for its competitive advantage rather than relying on landfills, which has thus far caused unsustainable MSWM.

Comparing the amount of CO_2_ emitted from composting and pyro-gasification of biodegradable waste, i.e., 0.09 and −0.56 MT of CO_2_/MT of treated waste, respectively, suggests that pyro-gasification is the better option. However, the economic cost for investing in pyro-gasification technology in Dar es Salaam makes it unacceptable. Instead, composting with its economic competitiveness and moderate environmental cost remains the best option for now. As suggested by scholars [111,112,113], composting organic matter will not only ameliorate the MSW problem in the city and other parts of the country but will also improve soil nutrients. This will add value to urban agriculture, which constitutes up to 45% of the economic activity of low-income urban dwellers in the city and its surroundings.

## 4. Conclusions

In this study, both economic and environmental costs for comprehensive MSWM were identified and presented. Each designed scenario had distinct features that differentiated it from others. Applying the ELECTRE method was useful for identifying the dominant scenarios in terms of reducing economic and environmental costs. The investigation considered the country’s economic, political, sociocultural, and policy aspects and pertinent international treaties on environmental standards. Of all the 108 explored cases, four (S19, S21, S25, and S20) met all the selection criteria. From these, S19 was found the most favorable, forming the core of the four considered. Thus, decision makers should first consider this scenario. The identified economic and environmental costs were estimated on a daily basis. This is seldom the case in current decision-making or in literature. The approach and the methods used are applicable to other cities with similar MSWM issues with or without adjusting the scenarios used in this study. They can also be used to determine the best methods of dealing with urban pollutants other than MSW, leading to sustainable environmental quality in developing countries.

## Figures and Tables

**Table 1:** Waste streams and potential treatment/management options.

Waste Stream/Treatment Options	Organic: Kitchen Waste, Market Waste, Garden Waste	Plastic and Rubber: Film, Rigid, other Related Plastic Waste	Paper: Paper and Paperboard	Glass	Metal: Steel, Aluminum	Others: Tetrapacks, Diapers, Non-Ferrous Metal
**1: Composting**	+	−	−	−	−	−
*Windrow composting*						
*Vermicomposting*						
**2: Recycling**	−	+	+	+	+	−
**3: Incineration with energy recovery**	−	+	+	−	−	+
**4: Pyro-gasification**	+	−	−	−	−	−
*Pyrolysis*						
*Gasification*						
*Anaerobic digestion*						
**5: Landfilling**	+	+	+	+	−	+
*Sanitary*						
*Bioreactor*						

**Table 2:** Average economic and environmental cost of municipal solid waste treatment options for all waste streams in Dar es Salaam.

**A: Economic Cost (USD/Metric Ton (MT))**								
**Waste Stream\Treatment Option**	**Organic Waste**	**Plastic Waste**	**Paper Waste**	**Glass**	**Metal**	**Other Waste**	**Country of Use**	**Sources**
1: Composting *Windrow composting* *Vermicomposting*	50.05	-	-	-	-	-	Australia, China, USA, South Africa (SA), Kenya, Ethiopia	[64,65,66,67]
2: Recycling	-	101.02	−45.20	20.50	18.56	-	China, Canada, USA, Nigeria, Taiwan, Japan, Wales	[58,65,68,69]
3: Incineration with energy recovery	-	20.00	20.00	-	-	55.05	India, China, England, Denmark, SA, Indonesia	[68,70,71,72,73]
4: Pyro-gasification *Pyrolysis* *Gasification* *Anaerobic digestion*	115.25	-	-	-	-	-	China, Nepal, UK, USA, Italy, Japan, Korea, India Pakistan, Iran	[70,74,75,76,77,78,79,80]
5: Landfilling *Sanitary* *Bioreactor*	58.25	71.10	67.25	70.32	-	68.33	Ethiopia, Morocco, USA, UK, Korea, China, Philippines	[64,65,68,72,75,77,81]
**B: Environmental Cost (CO_2_/MT)**								
**Waste Stream\Treatment Option**	**Organic Waste**	**Plastic Waste**	**Paper Waste**	**Glass**	**Metal**	**Other Waste**	**Country of Use**	**Sources**
1: Composting *Windrow composting* *Vermicomposting*	0.092	-	-	-	-	-	National and international organizations, including the United Nation (UN) in numerous environmental assessment and management programs	[53,64,65,71,82,83,84,85,86]
2: Recycling	-	−1.306	–3.893	−0.307	–3.38	-		
3: Incineration with energy recovery	-	1.377	–0.490		-	1.35		
4: Pyro-gasification *Pyrolysis* *Gasification* *Anaerobic digestion*	−0.560	-	-	-	-	-		
5: Landfilling *Sanitary* *Bioreactor*	0.474	0.043	0.441	0.444	-	0.477		

**Table 3:** Classification guide for the selection of acceptable scenarios.

Cost Level	EcC (USD/Day)	EnC (MT. CO_2_) Emissions/Day
Low	*y* = (210,000–240,000)	*x* ≤ −1000
Medium	*y* = (250,000–280,000)	*x* = −999–0
High	*y* = (290,000–300,000)	*x* = 1–360

EcC: economic costs; EnC: environmental costs.

**Table 4:** Average municipal solid waste generated in Dar es Salaam and composition by waste stream from 2006–2017 (MT/day).

Year	Amount Generated	Organic (57.21%)	Plastic (13.08%)	Paper (6.12%)	Glass (2.32%)	Metal (1.02%)	Others (20.25%)	Total (100%)
2006	3930	2248.35	514.04	240.52	91.18	40.09	795.83	3930.00
2007	4115	2354.19	538.24	251.84	95.47	41.97	833.29	4115.00
2008	4286	2452.02	560.61	262.30	99.44	43.72	867.92	4286.00
2009	4454	2548.13	582.58	272.58	103.33	45.43	901.94	4454.00
2010	4577	2618.50	598.67	280.11	106.19	46.69	926.84	4577.00
2011	4669	2671.13	610.71	285.74	108.32	47.62	945.47	4669.00
2012	4397	2515.52	575.13	269.10	102.01	44.85	890.39	4397.00
2013	4661	2666.56	609.66	285.25	108.14	47.54	943.85	4661.00
2014	4605	2634.52	602.33	281.83	106.84	46.97	932.51	4605.00
2015	4573	2616.21	598.15	279.87	106.09	46.64	926.03	4573.00
2016	4740	2711.75	619.99	290.09	109.97	48.35	959.85	4740.00
2017	5258	3008.10	687.75	321.79	121.99	53.63	1064.75	5258.00
Total	54,265	31,045.01	7097.86	3321.02	1258.95	553.50	10,988.66	54,265.00

**Table 5:** Generated scenarios and their coding.

Scenario	Code	Scenario	Code	Scenario	Code	Scenario	Code	Scenario	Code	Scenario	Code
S1	122223	S19	122225	S37	422223	S55	422225	S73	522223	S91	522225
S2	132223	S20	132225	S38	432223	S56	432225	S74	532223	S92	532225
S3	152223	S21	152225	S39	452223	S57	452225	S75	552223	S93	552225
S4	122523	S22	122525	S40	422523	S58	422525	S76	522523	S94	522525
S5	132523	S23	132525	S41	432523	S59	432525	S77	532523	S95	532525
S6	152523	S24	152525	S42	452523	S60	452525	S78	552523	S96	552525
S7	123223	S25	123225	S43	423223	S61	423225	S79	523223	S97	523225
S8	133223	S26	133225	S44	433223	S62	433225	S80	533223	S98	533225
S9	153223	S27	153225	S45	453223	S63	453225	S81	553223	S99	553225
S10	123523	S28	123525	S46	423523	S64	423525	S82	523523	S100	523525
S11	133523	S29	133525	S47	433523	S65	433525	S83	533523	S101	533525
S12	153523	S30	153525	S48	453523	S66	453525	S84	553523	S102	553525
S13	125223	S31	125225	S49	425223	S67	425225	S85	525223	S103	525225
S14	135223	S32	135225	S50	435223	S68	435225	S86	535223	S104	535225
S15	155223	S33	155225	S51	455223	S69	455225	S87	555223	S105	555225
S16	125523	S34	125525	S52	425523	S70	425525	S88	525523	S106	525525
S17	135523	S35	135525	S53	435523	S71	435525	S89	535523	S107	535525
S18	155523	S36	155525	S54	455523	S72	455525	S90	555523	S108	555525

**Table 6:** Economic and environmental costs of comprehensive municipal solid waste treatment for all scenarios.

Scenario	Code	EcC (USD/Day)	EnC (CO_2_/Day)	Scenario	Code	EcC (USD/Day)	EnC (CO_2_/Day)	Scenario	Code	EcC (USD/Day)	EnC (CO_2_/Day)	Scenario	Code	EcC (USD/Day)	EnC (CO_2_/Day)
												S80	533223	257,416.02	3433.89
												S81	553223	292,560.05	2516.43
				S26	133225	246,889.48	1355.27	S53	435523	450,159.84	714.71	S82	523523	311,649.82	1680.27
				S27	153225	282,033.51	437.81	S54	455523	485,303.86	−202.75	S83	533523	263,493.56	3525.50
S1	122223	259,925.15	−655.49	S28	123525	301,123.28	−398.35	S55	422225	470,193.15	−3546.30	S84	553523	298,637.59	2608.04
S2	132223	211,768.89	1189.74	S29	133525	252,967.02	1446.88	S56	432225	422,036.89	−1701.07	S85	525223	320,776.85	1888.24
S3	152223	246,912.92	272.28	S30	153525	288,111.05	529.42	S57	452225	457,180.92	−2618.53	S86	535223	272,620.60	3733.47
S4	122523	266,002.69	−563.88	S31	125225	310,250.31	−190.38	S58	422525	476,270.69	−3454.69	S87	555223	307,764.62	2816.01
S5	132523	217,846.43	1281.36	S32	135225	262,094.06	1654.85	S59	432525	428,114.43	−1609.45	S88	525523	326,854.39	1979.85
S6	152523	252,990.46	363.90	S33	155225	297,238.08	737.39	S60	452525	463,258.46	−2526.91	S89	535523	278,698.14	3825.09
S7	123223	280,905.86	439.56	S34	125525	316,327.85	−98.77	S61	423225	491,173.86	−2451.25	S90	555523	313,842.16	2907.63
S8	133223	232,749.60	2284.79	S35	135525	268,171.60	1746.47	S62	433225	443,017.60	−606.02	S91	522225	298,731.45	−435.93
S9	153223	267,893.63	1367.33	S36	155525	303,315.62	829.01	S63	453225	478,161.63	−1523.47	S92	532225	250,575.19	1409.31
S10	123523	286,983.40	531.17	S37	422223	456,053.27	−2616.77	S64	423525	497,251.40	−2359.63	S93	552225	285,719.22	491.85
S11	133523	238,827.14	2376.41	S38	432223	407,897.01	−771.54	S65	433525	449,095.14	−514.40	S94	522525	304,808.99	−344.31
S12	153523	273,971.17	1458.95	S39	452223	443,041.04	−1689.00	S66	453525	484,239.17	−1431.86	S95	532525	256,652.73	1500.92
S13	125223	296,110.43	739.15	S40	422523	462,130.81	−2525.16	S67	425225	506,378.43	−2151.66	S96	552525	291,796.76	583.46
S14	135223	247,954.18	2584.38	S41	432523	413,974.55	−679.93	S68	435225	458,222.18	−306.43	S97	523225	319,712.16	659.13
S15	155223	283,098.20	1666.92	S42	452523	449,118.58	−1597.38	S69	455225	493,366.20	−1223.89	S98	533225	271,555.90	2504.36
S16	125523	302,187.97	830.76	S43	423223	477,033.98	−1521.72	S70	425525	512,455.97	−2060.05	S99	553225	306,699.93	1586.90
S17	135523	254,031.72	2675.99	S44	433223	428,877.72	323.51	S71	435525	464,299.72	−214.81	S100	523525	325,789.70	750.74
S18	155523	289,175.74	1758.53	S45	453223	464,021.75	−593.95	S72	455525	499,443.74	−1132.27	S101	533525	277,633.44	2595.97
S19	122225	274,065.03	−1585.02	S46	423523	483,111.52	−1430.11	S73	522223	284,591.57	493.60	S102	553525	312,777.47	1678.52
S20	132225	225,908.77	260.21	S47	433523	434,955.26	415.13	S74	532223	236,435.31	2338.83	S103	525225	334,916.73	958.71
S21	152225	261,052.80	−657.24	S48	453523	470,099.29	−502.33	S75	552223	271,579.34	1421.38	S104	535225	286,760.48	2803.95
S22	122525	280,142.57	−1493.40	S49	425223	492,238.55	−1222.14	S76	522523	290,669.11	585.22	S105	555225	321,904.50	1886.49
S23	132525	231,986.31	351.83	S50	435223	444,082.30	623.10	S77	532523	242,512.85	2430.45	S106	525525	340,994.27	1050.33
S24	152525	267,130.34	−565.63	S51	455223	479,226.32	−294.36	S78	552523	277,656.88	1512.99	S107	535525	292,838.02	2895.56
S25	123225	295,045.74	−489.97	S52	425523	498,316.09	−1130.52	S79	523223	305,572.28	1588.65	S108	555525	327,982.04	1978.10

**Table 7:** Acceptable scenarios for initial decision natrix.

Scenario	Code	Economic Cost (USD/Day)	Environmental Cost (CO_2_ Emission; MT/Day)
S19	122225	274,065.03	−1585.02
S22	122525	280,142.57	−1493.40
S21	152225	261,052.80	−657.24
S1	122223	259,925.15	−655.49
S24	152525	267,130.34	−565.63
S4	122523	266,002.69	−563.88
S25	123225	295,045.74	−489.97
S91	522225	298,731.45	−435.93
S20	132225	225,908.77	260.21
S3	152223	246,912.92	272.28
S23	132525	231,986.31	351.83

**Table 8:** Groups of acceptable scenarios.

Group	EcC	EnC	Scenarios	Representative Scenario (Scenario with Least EnC)
Most favorable	Low/Medium	Low	S19, S22	S19
Favorable	Medium	Low/Medium	S21, S1, S24, S4	S21
Less favorable	High	Low/Medium	S25, S91	S25
Intermediate	Low	High	S20, S3, S23	S20
